# Supplementary material for: Are carbon emissions trading and green financial instruments synergistic? -Comprehensive quantitative research based on content analysis
Source: PLoS One. 2024 Mar 7;19(3):e0298601. doi: 10.1371/journal.pone.0298601 (PMC10919601; doi:10.1371/journal.pone.0298601)
Supplement: S1 Data — (ZIP) [file pone.0298601.s001.zip › Data/地级市Z_F工作报告.zip!/╔╜╢1⁄2/═■║ú╩╨/└┤╫╘╣1⁄2╓┌║┼í╛┬φ┐╦╩2╛▌═°í┐_2008.docx]

各位代表：

现在，我代表威海市人民政府向大会做工作报告，请予审议，并请各位政协委员和其他列席人员提出意见。

一、过去五年工作回顾

五年来，在中共威海市委的正确领导下，本届政府团结带领全市人民，坚持以邓小平理论和“三个代表”重要思想为指导，深入贯彻落实科学发展观，解放思想，抢抓机遇，求真务实，开拓进取，圆满完成了市十四届人大确定的各项目标任务。过去的五年，是大改革、大开放、大发展的五年，是社会全面和谐进步、人民生活显著改善的五年，是加快科学发展、打造人居福地，在建设全面小康和现代化新威海道路上阔步前进的五年。

――综合实力不断提升，科学发展成效显著。在结构优化、效益提高、能耗下降、生态改善的基础上，胜利完成了“十五”计划，“十一五”规划进展顺利，预计2007年全市生产总值达到1585亿元，年均增长16.8%。财政总收入达到156亿元，年均增长24.6%；其中地方财政收入82亿元，占生产总值比重由4.2%提高到5.2%。三大基地、五大产业群、四大支柱产业协调发展，三次产业比重由13.6:55.3:31.1调整为7.7：62.1：30.2。现代制造业取得新进展，五大产业群占规模以上工业增加值的比重由79.5%提高到83.7%，销售收入过亿元的工业企业从276家发展到761家。海洋经济初步实现了“四个转变”，海洋产业增加值占生产总值的比重由23.7%提高到26.9%。服务业不断升级，新型流通业态和现代营销方式所占比重由19.7%提高到42%，旅游总收入年均增长18.7%，社会消费品零售总额年均增长15.9%。固定资产投资年均增长41.3%，投资率由27.7%提高到47.3%。万元生产总值能耗、水耗分别降低30%和20%，化学需氧量和二氧化硫排放量分别削减4.7%和6.3%。

――改革开放不断深化，发展活力全面增强。企业产权制度改革基本完成，国有资产监管体制进一步理顺。上市工作取得历史性突破，10家企业成功上市。金融生态环境显著改善，新增贷款370.4亿元，年均增长15.8%。民营经济快速发展，新增个体业户和私营企业1.7万家，非公有制经济增加值占生产总值比重由40%提高到60.5%。参与国际经济竞争的能力进一步提高，累计实际利用外资58.9亿美元、内资630亿元。外贸进出口总值达到110亿美元，年均增长30.4%，机电产品出口比重由28.6%提高到45.6%。各类开发区在整顿中快速发展，创办了威海工业园、石岛管理区，启动了科技新城、临港工业园等新区的开发，打造了一批带动经济增长的新板块。

――自主创新能力不断增强，发展方式加快转变。大力推进了创新型城市建设，与山东大学、哈尔滨工业大学、中国海洋大学签订了产学研战略联盟合作协议。建立完善了科技创新平台和服务体系，省级工程技术研究中心和企业技术开发中心由24家发展到78家，国家级由2家发展到5家。培植壮大了一批创新型企业，规模以上工业企业研发投入占销售收入的比重由0.37%提高到0.56%，高新技术产业产值占工业总产值的比重由14.7%提高到30%。建设国家863计划成果产业化基地5个，取得重要科技成果475项，其中7项获得国家科学技术进步奖。新增19个中国名牌产品、7个中国驰名商标。引进各类人才3.1万人。科技和人才对经济发展的支撑作用明显增强。

――新农村建设不断深入，农村面貌发生深刻变化。农村综合改革逐步深化，农业税、特产税全部取消，支农惠农政策得到全面落实。财政惠农资金达到57.4亿元，占财政支出的比重由11.1%提高到16.9%。农业综合生产能力逐年提高，粮油产量保持稳定，粮经面积比由66：34调整到62：38。产业化和标准化程度进一步提高，销售收入过亿元的农业龙头企业由23家增加到66家。加强了“路水电气医学”建设，基本实现了村村通油路、通客车、通自来水、通有线电视，建设户用沼气1.3万座，新型农村合作医疗覆盖面达到96.9%，农村省市级规范化学校建成率达到90%，培训转移农村劳动力10.4万人。

――基础设施不断完善，城市化水平有新的提高。实施了“公路升级、铁路提速、港口扩建、机场开放”战略，公路通车里程由2405公里增加到6604公里；铁路客运量由112万人次增加到219万人次；港口吞吐量由916万吨增加到3180万吨；威海机场成功对外开放，旅客吞吐量由5万人次增加到62万人次。修编了新一轮城市总体规划和108项详细规划，拉开了一体化威海建设的大框架，建成了幸福公园等一批精品工程，城市化率由42%提高到57%，中心城市建成区面积由44平方公里扩大到109平方公里。荣成市、文登市、乳山市均启动了新城区建设规划，城市道路交通进一步优化，供水、供气、供热和污水处理能力显著提高。全市森林覆盖率由29%提高到37%，建成区绿化覆盖率由39.8%提高到46.3%。先后成为全国第一个“国家环境保护模范城市”群、“中国优秀旅游城市”群、全省第一个“园林城市”群，荣获“联合国人居奖”、“全国绿化模范城市”、“中国人居环境奖”、“全国节水型城市”等称号。

――收入水平不断提高，人民群众得到更多实惠。城镇居民人均可支配收入和农民人均纯收入年均增长11.5%和11.9%，分别达到16211元和7730元。城乡居民人均储蓄存款年均增长12.3%，达到22693元。城市居民人均居住使用面积由14.7平方米增加到18.8平方米，农村居民人均住房面积由27平方米增加到34平方米。移动电话普及率由16.6%提高到63.5%，宽带用户由11万户发展到26万户。家庭轿车由1.4万辆发展到6.4万辆。城乡居民的恩格尔系数降到29.4%和36.6%，消费结构发生新的变化。

――和谐社会建设不断推进，各项社会事业全面进步。免除了义务教育阶段的学杂费，建立了经济困难家庭学生财政资助体系，中小学办学条件得到明显改善。职业技术教育迅猛发展，引进和扩建了5所高等职业学院，高校全日制在校生由1.2万人增加到4.5万人。就业规模不断扩大，城镇登记失业率控制在1.6%以内。社会保障体系进一步健全，建立了城镇居民基本医疗保险和城镇合作医疗制度，职工养老、医疗保险参保人数分别扩大0.6倍和0.9倍。以政府为主导的新型社会救助体系更加完善，城乡低保和五保供养标准大幅提高，65处镇级敬老院全部改造升级，新建了13所老年公寓、儿童福利院，建设经济适用房和廉租房84.9万平方米，困难群众生活得到及时有效救助。建立了独生子女父母奖励费统一发放和计划生育困难家庭救助制度。农村文化大院、城市广场文化等群众性文化体育活动日益活跃，广播电视、新闻出版、文物保护事业得到加强，史志、档案资源开发利用取得新成果。国防动员、人民防空、民兵预备役工作迈出新步伐，连续五次荣获“全国双拥模范城”称号。妇女儿童、老年人、残疾人权益得到较好保障。外事、侨务、涉台、民族、宗教、仲裁、气象、地震等事业都有新发展。民主法制建设得到加强，“平安威海”建设成效显著，跨入“全国社会治安综合治理优秀地市”行列。“文明威海”、“诚信威海”建设扎实推进，荣获“创建全国文明城市工作先进城市”称号。

――政府自身建设不断加强，行政服务环境得到优化。深入开展了解放思想大讨论等活动，加强了公务员培训和考核。深化了行政管理体制改革，建立健全了市、县、镇三级行政审批服务体系，市级精减审批事项331项，审批时限平均缩短80%。推进了部门预算编制、国库集中支付、政府采购、县乡财政体制、公务用车货币化和公务员分配制度改革，健全了国有土地、社保基金、住房公积金监管制度，加强了经济责任审计，进一步规范了行政行为。建立健全了应急管理体系，成功应对了非典、禽流感、风暴潮和百年一遇暴风雪等突发事件。开通了“12345”市长公开电话和电子信箱，开办了行风热线和直播威海栏目，成立了社情民意调查中心，加强了民主评议行风工作。办理了281件人大代表建议、679件政协委员提案，邀请了部分人大代表、政协委员及市民列席市政府重要会议，完善了重大事件听证制度。认真履行“一岗双责”，坚持从源头上反腐倡廉，严肃查处了一批违法违纪案件，政风行风都有新的转变。

各位代表！刚刚过去的一年，市政府认真贯彻落实党的十七大和省九次党代会、市十三次党代会精神，紧紧围绕市委提出的争当“三个发展”排头兵的目标，按照科学发展观的要求，用力推进现代制造业、海洋经济、现代服务业、能源石化等重点产业的发展，并以大项目建设和骨干企业膨胀为总抓手，推动了经济社会又好又快发展。预计全市生产总值增长15.5%左右；财政收入实现了总量质量双提高，地方财政收入增长17%左右，税收占地方财政收入的比重提高了3个百分点左右；万元生产总值能耗降低4.5%、水耗降低4%，化学需氧量和二氧化硫排放量分别削减7.5%和1.5%；固定资产投资增长22%左右；社会消费品零售总额增长17.8%左右；外贸出口增长16%左右，外商直接投资增长7.2%左右，实际利用内资增长15%左右；城镇居民人均可支配收入增长16%左右，农民人均纯收入增长13%左右。99项重点工作和为民办的9件实事基本完成，主要指标达到或超过预期目标。

各位代表！回首过去的五年，我们在科学发展、和谐发展、率先发展的道路上取得了丰硕的成果。成绩的取得，是市委总揽全局、掌舵把关、撑腰鼓劲、坚强领导的结果，是市人大及其常委会寓支持于依法监督之中、积极开展有效工作监督的结果，是市政协寓支持于民主监督之中、积极参政议政的结果，是市纪委、法院、检察院保驾护航、监督支持的结果，是驻威部队官兵携手共建、无私奉献的结果，是社会各界朋友竭诚尽智、献策献力的结果，是各级、各部门和全市人民齐心合力、埋头苦干的结果。在此，我代表市人民政府，向全体市民和所有纳税人，向各位人大代表和政协委员，向各民主党派、工商联、各人民团体和社会各界人士，向驻威部队指战员、武警官兵和政法干警，向各位老领导、老同志，向所有关心支持威海发展的海内外朋友，致以崇高的敬意和衷心的感谢！

各位代表！回顾过去的工作，我们深切体会到，要不断开创改革开放和全面建设小康社会的新局面，必须坚定不移地与党中央保持高度一致，坚决贯彻执行省委、市委决议，创造性地开展工作；必须坚持解放思想、与时俱进，用新思维、新观念、新举措应对新形势、新情况、新问题；必须坚定不移地推进改革开放，建立完善充满生机活力的新体制、新机制，不断解放和发展社会生产力；必须牢牢抓住第一要务，坚持以人为本，统筹兼顾，努力实现全面协调可持续发展；必须牢记全心全意为人民服务的宗旨，多为群众办好事、办实事，让人民共建共享和谐社会；必须坚持真抓实干，以求真务实的作风推进各项工作，多干打基础、利长远的事，以实干实效赢得群众的信赖和支持。

在总结成绩的同时，我们也清醒地看到面临的困难和问题：生产力水平与质量、效益还不够高，自主创新能力还不够强，增长方式仍然比较粗放；经济结构还不够优化，大项目少、骨干企业不强的矛盾仍比较突出，现代服务业发展还相对滞后；部分行业、企业能源消耗、污染排放仍然偏高，资源节约、生态环境保护的压力依然较大；开放型经济层次不够高，制约发展的体制机制障碍在一些领域依然存在；农民增收的困难加大，统筹城乡发展，缩小城乡差距任重道远；与发达地区相比人民群众的富裕程度还不够高，社会民生方面仍有不少亟待解决的问题；工作作风、行政效能与发展要求还有一定差距，依法行政的能力和水平有待进一步提高，廉政建设和行风建设还需要进一步加强，等等。对此，我们一定保持清醒认识，增强忧患意识，采取有力措施，认真加以解决。

二、今后五年政府工作的目标任务

各位代表！过去的五年成绩显著，未来的五年任重道远。党的十七大确立了到2020年全面建成小康社会的奋斗目标。省九届二次全委会提出了“一体两翼”区域发展战略。市十三届二次全委会确定了我市争当“三个发展”排头兵的战略目标：到2012年，人均生产总值比2000年翻两番，到2020年翻三番。围绕上述目标，今后五年政府工作的总体要求是：全面贯彻党的十七大精神，高举中国特色社会主义伟大旗帜，以邓小平理论和“三个代表”重要思想为指导，深入贯彻落实科学发展观，坚决执行国家宏观调控政策，认真落实省委、省政府和市委的各项决策部署，坚持好字优先，紧紧围绕转变经济发展方式和完善社会主义市场经济体制，积极推进改革开放和自主创新，着力优化经济结构和提高经济发展质量，切实加强节能减排和生态环境保护，大力推进世界精品城市建设，更加重视改善民生和促进社会和谐，积极融入全省“一体两翼”区域发展格局，努力推动科学发展、和谐发展、率先发展。今后五年的主要目标任务是：

――综合实力大幅提升。坚持好中求快，着力把握发展规律、创新发展理念、转变发展方式、破解发展难题，实现又好又快发展。按照全面对接、整体融入、突出特色、形成优势的原则，加快与半岛城市群的一体化发展，努力在“一体两翼”区域发展中发挥更大的作用。全市生产总值年均增长15%以上，人均达到10.6万元；地方财政收入年均增长17%，效益指标要明显高于生产指标的增长，主要经济指标人均占有保持全省领先水平。

――人民生活走向富裕。坚持以人为本、富民优先，建立健全促进城乡居民持续增收的长效机制，逐步提高居民收入在国民收入分配中的比重、劳动报酬在初次分配中的比重、财产性收入在居民收入中的比重，扩大中等收入者比重，提升小康生活的质量。完善农村基础设施，搞好绿化美化，发展公益事业，推动新农村建设迈出坚实步伐。稳定粮食面积，发展经济作物，提高农业综合生产能力，确保主要农副产品有效供给。城镇居民人均可支配收入年均增长10%以上，农民人均纯收入年均增长8%以上。

――发展方式明显转变。加快推进需求结构、产业结构、要素结构“三个转变”，促进经济增长由主要依靠投资、出口拉动向依靠消费、投资、出口协调拉动转变，由主要依靠第二产业带动向三次产业协同带动转变，由主要依靠增加物质资源消耗向主要依靠科技进步、劳动者素质提高和管理创新转变，增强发展的稳定性和协调性，三次产业比重调整为5：59：36。加快推进三大基地、五大产业群建设，突出抓好现代制造业、海洋经济、现代服务业、能源石化四大支柱产业，高新技术产业产值占工业总产值的比重提高10个百分点。大力推进创新型城市建设，各类专业技术人员占总人口比重达到7.9%，全市研发投入占生产总值比重达到1.75%。突出抓好大项目建设和骨干企业膨胀，力争有10家工业企业销售收入过50亿元，其中7家过100亿元；流通企业5家过10亿元，其中1家过100亿元。

――改革开放深入推进。加快推进重要领域和关键环节的改革，进一步深化行政管理体制改革、事业单位改革和企业改革，建立健全有利于科学发展的体制机制。加强金融生态环境建设，大力推进银企合作，推动优势骨干企业上市。推进公平准入，改善融资条件，破除体制障碍，使民营经济真正成为主体经济、富民经济。进一步突出开放的龙头地位，提高开放型经济水平。外商直接投资和外贸进出口分别年均增长8%和12%以上。

――基础设施更加完善。大力实施“兴港强市”战略，以发展客运、集装箱和液体码头为重点，强化中心港地位，加快港口资源整合，新建扩建35个万吨级以上泊位，新开10条客货集装箱班轮航线，港口吞吐量过亿吨，集装箱吞吐量达到150万标准箱。争取开工建设烟威荣石铁路，完成威海机场和桃威铁路改扩建工程，机场旅客吞吐量达到100万人次，铁路客运量达到500万人次、货运量达到200万吨。建成荣乌高速公路荣成至文登段、滨海旅游景观公路，争取开工建设荣潍高速公路文登至莱阳段。

――生态环境更加优化。森林覆盖率每年增加2个百分点，城市污水集中处理率提高到85%以上，功能区水质达标率达到100%。万元生产总值能耗降低到0.7吨标准煤、水耗降低到18立方米，化学需氧量和二氧化硫排放量分别削减5.3%和2.2%。坚持生产发展、生活富裕、生态良好的文明发展道路，建设资源节约型、环境友好型社会，生态文明观念在全社会牢固树立。按照“优化开发”的主体功能区定位，调整优化经济布局，促进国土资源集约有序开发，提高资源环境承载能力。扎实推进节能减排体系建设，强化对重点用能企业和重点排污企业的监管，确保完成约束性指标，在全省率先建成国家生态市。

――精品城市更加亮丽。中心城市建成区面积扩大到125平方公里，人口达到100万人，城市化率达到60%以上。继续坚持高起点规划、高标准建设、高效能管理，建设精品城市。按照城乡一体化的原则，加强中心城市与次中心城市、中心镇的规划衔接，优化城乡空间布局。强化精品意识，不断提高新建城区的档次和品位。按照一次规划、分步实施的原则，进一步扩大水、电、气、暖等基础设施的覆盖面，建设完善高效的城市基础设施体系。市区自来水、燃气基本普及，集中供热率达到72%。调整完善道路功能层次和路网空间形态，在重点地区、重点地段、住宅区和大型公共设施周边规划建设一批停车场、过街天桥和地下通道，妥善解决交通堵塞和停车难问题。加快城中村改造步伐，用三年时间完成市区主要城中村改造。

――文化事业繁荣发展。文化产业增加值占生产总值的比重达到3%，初步形成比较完备的文化产业和市场体系。把文化建设摆上突出位置，推动社会主义文化大发展大繁荣。坚持社会主义核心价值体系，加强思想道德建设，大力弘扬“威海精神”，深入挖掘传统文化、特色文化，大力繁荣和谐文化、人居文化、幸福文化，不断提升威海的软实力。加大对公益性文化事业的投入力度，强化公共文化设施建设，高标准建设好市民文化中心、全民健身中心和健身广场。支持发展文化产业，构建4个示范基地，打造动漫产业、旅游文化基地和演艺品牌。

――社会建设和谐有序。按照十七大提出的“五有”目标，更加注重社会建设，完善社会管理，扩大公共服务，着力保障和改善民生，促进社会公平正义。免费义务教育制度更加完善，城乡教育均衡发展、各类教育协调推进。城乡居民实现充分就业，城镇登记失业率控制在2%以内。收入分配更加合理有序，社会保障体系更加健全，城镇职工人人享有基本养老保险，城乡最低保障覆盖面达到3%以上。覆盖城乡居民的基本医疗卫生保健制度基本建立，城镇居民医疗保险基本实现全覆盖，新型农村合作医疗的保障水平显著提高。五保对象集中供养率达到75%，敬老院实现愿进全进。经济适用房和廉租房保障范围进一步扩大，基本解决人均住房建筑面积10平方米以下低收入家庭的住房困难。民主法制建设更加完善，三市一区进入省级村民自治先进市（区）行列，达到省“平安县（市区）”标准，确保社会稳定和人民群众生命财产安全。

各位代表，上述目标任务的确定，与“十一五”规划进行了衔接，充分考虑了全面建设小康社会和争当“三个发展”排头兵的需要，是积极稳妥的。我们将在省委、省政府和市委的坚强领导下，团结带领全市人民埋头苦干，奋发拼搏，努力把宏伟蓝图变成美好现实，让广大群众在科学发展中共创大业，共享和谐！

三、2008年政府工作重点

2008年是深入贯彻党的十七大精神、认真落实全省“一体两翼”区域发展战略的第一年，是新一届政府的起步年。按照中央经济工作会议和省九届三次全委会、市十三届三次全委会精神，初步确定今年经济社会发展的主要预期目标为：生产总值增长15%，地方财政收入增长17%，万元生产总值能耗、水耗分别降低4.5%，化学需氧量和二氧化硫排放量分别削减3.2%和0.5%，固定资产投资增长18%，社会消费品零售总额增长17%，外商直接投资增长10%，实际利用内资增长10%，外贸出口增长15%，城镇居民人均可支配收入增长11%，农民人均纯收入增长9%，城镇登记失业率控制在2%以内，人口自然增长率控制在1‰以内。围绕上述目标，在新的一年工作中，我们要把握好“稳”与“进”、“保”与“压”、“好”与“快”的关系，坚持稳中求进、好字优先，按照“控总量、稳物价、调结构、促平衡”的要求，抓好“两个防止”，促进经济社会又好又快发展。重点抓好以下十项工作：

（一）全方位搞好对接，积极融入全省“一体两翼”区域发展格局。省委、省政府提出的“一体两翼”区域发展战略，是新时期实现新跨越的战略举措，我们必须从思想上高度重视，把威海的发展置于全省的大局中统筹考虑，按照“着力推进优化开发，努力构建现代产业体系，增强核心竞争力”的要求，从规划、产业、基础设施、人才、合作机制等方面搞好对接。一是规划对接。遵循一体化和协同发展的原则，打破行政区划界限，加快城市各类规划建设与半岛城市群的对接。二是产业对接。按照“立足特色、错位竞争”的原则，调整优化产业发展重点，主动接受辐射，鼓励发展造船、汽车、电子信息等配套产业，培植“小件巨人”，增强与周边城市产业的关联性和互补性。推动与周边城市现代服务业的融合发展，加强旅游合作，打造“黄金海岸”线。三是基础设施对接。以实现全方位交通联网为目标，实现与周边城市的高速连接。加快推进荣乌高速公路荣成至文登段建设，做好荣潍高速公路文登至莱阳段、烟威荣石铁路的争取工作，加强威海港与青岛港的合作。四是人才对接。认真落实青烟威三市共同签署的胶东半岛制造业基地人才开发一体化工作机制合作协议，引导企业加强与省内科研机构、大专院校的产学研合作，特别是加强与济、青、烟大专院校的合作。五是合作机制对接。与有关市加强沟通联系，建立完善工作交流机制，实现信息资源共享，在各项政策上全面衔接，避免无序竞争，形成一体化发展的合力。

（二）着力转变经济发展方式，加快推进重点产业发展。坚持把大项目建设作为转变经济发展方式的重中之重，按照“支持现有骨干企业膨胀一批、扶持潜力项目壮大一批、抓好招商引资培育一批”的思路，推动重点项目建设和骨干企业膨胀，带动四大支柱产业快速发展。一是加快发展现代制造业。大力推进信息化与工业化融合，着力培植一批骨干企业、重点产业和知名品牌，工业增加值增长15%。落实优惠政策，优化发展环境，把培植销售收入过百亿元、上缴税金过5亿元的企业作为膨胀骨干企业的重中之重来抓，争取新增过5亿元的企业20家，其中过10亿元的5家。大力扶持重点产业，抓好22个重点工业项目建设，进一步壮大五大产业群，争取在造船、汽车、装备制造业、电子信息、新材料、食品医药产业等方面实现新突破。鼓励中小企业与大企业开展协作配套，拉长产业链，促进产业集群和企业集群发展。二是大力发展现代服务业。坚持把服务业作为优化经济结构、培植地方财源的重点来抓，加大对省政府确定的4个重点城区、3个重点园区和5家重点企业的扶持力度，力争服务业增加值比重达到32%，提高2个百分点；提供的地方税收增长25%。要按照上年度全市GDP的万分之零点五的比例，安排服务业发展引导资金，做大做活“六大产业”。（1）文化旅游业，围绕旅游总收入增长15%以上的目标，坚持以千公里海岸线为载体，以福文化为主线，以项目建设为重点，抓好锦湖韩亚高尔夫、海光旅游开发及主题公园、天沐温泉度假村、大乳山福地养生园、仙姑顶希望如意园、威海湾海上旅游、圣经山综合开发、文登香水海等投资过亿元的重点旅游项目，推动由点式开发向串珠成链转变。加强宾馆等接待设施建设，新开工星级酒店5家。强化旅游促销宣传，逐步推进旅游业由观光型向休闲度假型转变。大力推进文化、旅游、会展等产业融合发展，抓好动漫产业发展基地、书画博览园、华夏城等重点项目建设。（2）商贸流通业，突出抓好乳山久久发国际商贸城、台资产品博览中心、九龙城、乐天利群购物中心等十大重点项目。（3）现代物流业，抓好中外运物流、中韩整车物流、中韩商品贸易中心、温泉物流园区、市区水产品批发市场和石岛北方渔市等项目建设。（4）房地产业，新开工建筑面积300万平方米，增长50%以上。完善房地产交易、中介、物业管理等服务，建立合理的住房供给体系，进一步扩大中小套型住房的供应量。（5）金融保险业，年末存、贷款余额要分别增长12%以上。加强金融生态环境建设，吸引国内外金融机构来威海落户，推进与国家开发银行、世界银行的合作项目，推动100个银企合作项目。（6）科技与信息服务业，信息服务业增加值增长25%。抓好信必优等服务外包项目，争取成为全省十大重点服务外包园区。三是突出发展现代海洋经济。加快推进海陆一体联动发展，力争海洋产业增加值占生产总值的比重达到28.9%，提高2个百分点。海洋一产，大力发展名优养殖和远洋渔业，新增无公害水产品产地3万亩，新增远洋渔船7艘，远洋捕捞产量达到9万吨。海洋二产，精深加工能力达到80万吨，比重达到75%，提高2个百分点；新增造船能力30万载重吨，力争总能力超过100万载重吨。推动水产品精深加工和造船业上规模、上档次，抓好5个重点水产品加工项目区，建设好皂埠湾、俚岛湾、石岛湾三大造船基地和新船重工、黄海造船、西霞口造船、三星重工、神飞船舶、三进船业、百步亭船业、乳山造船等十大造船企业。海洋三产，新增万吨级泊位6个，港口吞吐量达到3500万吨，集装箱吞吐量达到57万标箱，分别增长10%和14%。加快发展临港物流、海上运输、滨海旅游等现代产业，完成威海港三四期工程，抓好国际客运中心建设，抓紧实施港口资源整合，统筹石岛港、龙眼港、张家埠港、蜊江港、乳山口港协调发展。四是集中突破能源石化产业。经过多年多方面努力，中石油镆铘岛石化基地项目有望落户。去年省政府和中石油签订了合作备忘录，我们一定要紧紧抓住机遇，加强领导，集中力量，全力以赴抓好立项工作。同时，抓好文登华岳丙烯项目，支持相关石化产业发展。加快推进红石顶核电站、石岛湾核电站、昆嵛山抽水蓄能电站等3个项目的前期工作，抓好华能、国华、鲁能、中海油等4个风电项目，年内新增风电装机规模5万千瓦，总规模达到13.3万千瓦。

（三）以产学研结合为重点，大力提高自主创新能力。把自主创新作为威海发展的核心战略支撑，深入开展“自主创新年”活动，认真落实中长期科技发展规划纲要和支持企业自主创新的各项政策，突破关键技术，加快成果转化，提高科技进步对经济增长的贡献率，高新技术产业产值占工业总产值的比重提高2个百分点，达到32%。一是大力推进产学研合作。加快建立以企业为主体、市场为导向、产学研相结合的技术创新体系，支持创新要素向企业集聚。引导企业加强与大专院校和科研机构合作，通过各种途径多举办各类产学研合作洽谈会。认真落实与山东大学、哈尔滨工业大学、中国海洋大学签订的产学研战略联盟合作协议，年内争取再与3家以上国内知名大学签订合作协议。建立联系分包制度，逐个落实与高校签订的科技合作项目。加强沟通协调，争取建立中科院威海综合技术转化中心及山大、哈工大威海技术转移中心。引导企业建立科研机构，新建省级以上工程技术研究中心3家、企业技术开发中心2家。二是强化科技创新投入。安排企业科技创新资金和科技三项经费5800万元，优化资金使用办法，重点引进科技含量高、市场前景好、竞争力强的高新技术大项目和高水平发明专利项目。发挥企业主体地位，完善科技投入体系，规模以上工业企业研发投入占销售收入的比重达到0.66%，全市研发投入占生产总值的比重达到1.23%，省级以上企业技术开发中心科技活动经费支出比重达到4%。组织实施180项市级以上科技计划，推进大型船舶开发与设计、高端医疗器械、软件与系统集成等10项关键技术研发。积极做好“新三板”试点的争取工作，支持高新技术企业多渠道融资。三是提高科技服务支撑能力。启动汽车电子、海洋技术开发等科技创新服务平台及孵化器建设，进一步优化和集成科技资源。推进国家知识产权试点城市、试点园区建设，鼓励发明填补国内国际空白的科技项目，加强节能环保、装备制造、安全生产等领域的重大技术科研公关，开发具有自主知识产权的核心技术，争取专利申请量增长10%以上。抓好50个国外智力引进项目，完善创新型人才引进培养机制，形成结构合理的创新人才梯队。

（四）着眼于提高层次和质量，不断提升开放型经济水平。力争利用外资15.4亿美元、内资285亿元。外贸进出口超过123亿美元，增长12%。一是加大招商引资力度。按照“以企为主、产业对接、力求实效”的原则，组织好赴韩国、美国、京津、沪浙等11次重点经贸合作活动，邀请韩国生产性本部、美国服务外包代表团、上海知识产权园考察团等50个重要团组前来洽谈，多邀请国有大型企业法人来威考察，多主动到国有大型企业去沟通，筛选100家经济实力较好的民营企业作为对外推介重点，促进与国内外大公司的合作。加强与长三角、珠三角、环渤海地区等重点区域的合作交流，拓展经济、科技、旅游、人才等领域合作的深度和广度。在抓好制造业引资的同时，推动物流、城市公用设施、文化、旅游、会展等服务领域的合资合作。二是加快转变外贸增长方式。立足以质取胜，扩大机电产品出口比重，调整优化进出口结构，推动加工贸易升级转型。建立市场开拓基金，重点支持自主品牌、自主知识产权产品、机电和高新技术产品、节能环保产品出口。充分发挥市农副产品检测中心的作用，健全产品安全质量体系，巩固和扩大水产品等传统产品出口。引导企业获得国际标准认证，扶持名牌企业和名牌产品，支持15家入选企业争创省级以上出口名牌。组织企业参加50个国内外展会。三是搞好开发区建设。充分发挥园区建设的龙头带动作用，要继续举全市之力抓好威海工业园发展，使其成为现代制造业发展的新载体、新亮点。加快推进高区科技新城、经区临港工业园、石岛管理区、文登南海等新兴园区建设，强化载体功能，提高配套水平，引导新上项目向园区集聚。四是支持企业“走出去”。积极开展国际能源资源互利合作，支持有条件的企业到国外承包工程和开发林业、矿产、渔业等资源。

（五）扎实推进新农村建设。以促进农民增收为核心，进一步落实各项惠农政策，增强农业和农村经济的发展活力。一是大力发展现代农业。决不放松粮食生产，有粮面积稳定在220万亩以上，产量稳定在80万吨以上。积极调整种植业结构，建成农业科技示范区和基地62万亩，新增农业标准化生产基地10万亩。大力发展畜牧业，努力保障主要农产品基本供给，肉、蛋、奶和特种皮毛产量分别增长3%、4%、8%和10%。强化农业科技成果转化和推广应用，推进农业机械化。积极支持农业产业化经营，加快培育农产品名牌，新增省级以上重点企业4家，发展农村专业合作组织50家。二是加强农村基础设施建设。改造农村公路403.9公里，解决449个村的通油路问题。完成171个村的自来水新建改造。新增沼气用户1.2万户、沼气工程23处。深入实施“万村千乡市场工程”，加快农村现代流通服务网络建设，新建农村社区综合服务中心10家、农家店300处。三是积极发展农村社会事业。加强镇文化站、农村文化大院建设，新建文明生态村260个。选择300个有场地条件的村，增设体育健身设施。继续抓好联系村、学校、敬老院活动，改善65所敬老院取暖、洗浴、娱乐等设施条件。

（六）加强节能减排和环境保护，积极推进生态市建设。坚持节约优先、环保优先，把推进现代化与建设生态文明有机统一起来，年内通过国家生态市验收。一是大力抓好节能降耗。严格执行国家政策，建立更加有效的节能、节水、节地、节材机制。严格实行节能评估，加强对45家重点用能企业的能耗监测和计量管理。深化资源性产品价格改革，对超耗能企业执行差别电价等政策。加强土地审批管理，严格落实投资强度、建筑容积率和密度标准。盘活现有存量土地和闲置资源，用好现有的115万平方米的闲置厂房。加强建筑节能管理，严格执行节能强制性标准。加快再生资源回收利用体系建设。二是强力推进治污减排。认真开展第一次全国污染源普查工作，加强对100家重点污染源的在线监测，抓好5家省强制性清洁生产企业的审核，威海热电厂等6家电厂全部完成脱硫工程，淘汰30%的立窑水泥生产能力。蓝星玻璃两年内完成搬迁。加快5个污水处理厂项目建设，搞好管网配套，重点提高开发区、中心镇的污水处理能力。建立饮用水源安全预警制度，对12个饮用水源地开展大规模的综合治理。加强机动车尾气等专项治理，完善城市生活垃圾处理收费制度。狠抓林木育苗，加快推进绿色通道、荒山荒滩绿化、镇村绿化、沿海防护林等工程建设，森林覆盖率力争提高2个百分点。

（七）加快推进精品城市建设，进一步提高城乡规划建设管理水平。一是统筹城乡规划修编。深入贯彻《城乡规划法》，进一步完善城乡统筹规划和城市总体规划，加快修编次中心城市、小城镇总体规划和80%村庄的建设规划，推进各类详细规划的编制，逐步实现城乡规划全覆盖。严格执行各类规划，依法维护规划的严肃性和权威性。二是搞好重点工程建设。牢固树立精品意识，把高水平规划、高标准建设贯穿于项目建设的始终。市区推进大操场改造、环翠楼公园改造、金线顶区域整体改造、小石岛区域海岸线整理等15项重点工程建设，荣成市抓好城市展览中心、绿岛湖开发等52个重点项目，文登市抓好城市新区、国际会展中心、贸易中心等59个重点项目，乳山市抓好火车站改造、汽车站搬迁等30个重点项目，进一步提高承载能力。三是抓好河道综合整治。市区年内投资1.1亿元，铺设管网72公里，完成望岛河、钦村河、涝台河等9条市区河道的整治改造任务。全面治理市域范围内的主要河流，清查各流域污染源，加快推进污水入网，搞好河道两岸卫生管理和生态美化，确保治理一条污染河道，建成一条生态景观带。四是加快推进城中村改造。年内拆迁1.2万户、125.3万平方米，新开工建筑面积213.7万平方米。严格落实补偿和安置标准，配置和完善学校、医院等公用设施，确保拆一个旧村建一个精品社区，方便、富裕一方百姓。五是改善城市综合交通。市区新建改造塔山中路等14条道路，推进半月湾和统一路隧道建设，新建2个停车场、3个过街天桥，新增30部公交车，新开3条公交线路，方便市民出行。六是扩大基础设施覆盖面。抓好米威调水净水厂、工业园污水处理厂等8项供排水重点工程，日供水和污水处理能力分别增长55%和60%。推进城市电网建设改造，完成初村新区等区域的燃气和供热管网建设，新增天然气用户5000户、热用户1.2万户。七是开展城乡环境综合整治。探索建立长效管理机制，抓好重点部位、城乡结合部和住宅小区的专项整治，完成市区光明、塔山、花园、新威、杏花村等5个小区的背街小巷整治，营造良好的城乡环境。

（八）以提高发展质量为重点，积极做好财税工作。深入推进财税体制改革，稳步实现质量总量双提高，地方财政收入占生产总值的比重、税收占地方财政收入的比重、工商税收占地方财政收入的比重、四税占地方财政收入的比重，按可比口径分别提高0.1、0.1、1和0.5个百分点。大力培植财源经济，完善税源监控体系，加强重点税种和重点行业的征收管理，做到依法治税，应收尽收，使经济发展的成果体现到财税增收上来。抓好招商引税工作，加快企业二三产业分离。进一步优化财政支出结构，严格控制一般性支出，新增财力重点向“三农”、社会事业、环境保护等方面倾斜，加快建设“民生财政”。

（九）进一步深化各项改革，切实增强发展活力。一是完善国有资产管理体制。健全负责人激励约束、重大事项监督管理、预算管理和收益管理机制，盘活调优做强国有资产，实现保值增值。二是深化企事业单位改革。加快推进事业单位分类改革，继续深化收入分配制度改革，完善新进人员公开招聘制度。抓好机关事业单位办实体的规范和清理，合理处置经营性资产。三是下力气推进企业上市融资。抓住深圳中小板加速扩容等有利时机，推进华东数控、新北洋、天润曲轴、三角等企业上市，力争好当家、威高、广泰空港等实现再融资，上市融资20亿元。四是加快发展民营经济。落实推进民营经济发展的25条措施，完善公共技术、融资担保、人才培训、信息咨询和行业协会等服务体系，加快实施小企业培育计划和特色产业提升计划，年内新发展私营企业1000家，新增规模以上民营企业100家。

（十）以改善民生为重点，全面推进和谐社会建设。一是办好人民满意教育。进一步巩固城乡免费义务教育的成果，对农村义务教育阶段学生免费提供教科书，保障经济困难家庭、进城务工人员子女平等接受义务教育，确保不让一个学生因贫困而辍学。统筹城乡教育发展，进一步改善农村办学条件，创建农村规范化学校11所。优化城区中小学布局，新建和扩建中小学4所，新增校舍4万平方米。大力发展职业技术教育，面向市场需求，加强重点专业建设，提高技能型人才培养质量。二是千方百计扩大就业。全面落实各项就业再就业政策，加快推进市中心人力资源市场建设，积极做好高校毕业生就业工作，及时帮助困难群体解决就业问题。年内新增就业4.5万人，培训高技能人才1500人，转移农村劳动力1.6万人。建立健全职工工资正常增长和支付保障机制，严格落实企业最低工资保障和工资指导线制度，今年将最低工资标准由610元提高到760元。三是健全社会保障体系。积极扩大基本保险覆盖面，城镇职工基本养老、医疗和工伤保险参保人数分别增加1.7万、2.5万和3.5万人，城镇居民基本医疗保险覆盖面达到70%以上，农民养老保险参保人数达到33万人。建立困难群众建档立卡、定期走访制度，确保应保尽保、应救尽救、应助尽助。完善城乡居民最低生活保障和失地农民养老保险制度。密切关注价格走势，加强价格监管，严格控制出台政府调价项目，控制居民消费价格过快增长，及时完善和落实因基本生活必需品价格上涨对低收入群众的补助办法，严厉打击制售假冒伪劣商品、扰乱市场秩序等不法行为，组织好重要商品、生活必需品的供应，确保不断档、不脱销。大力发展老龄事业，抓好公共养老设施建设，农村五保集中供养率达到72%以上。大力发展慈善事业，加强困难群众医疗、教育、住房等专项救助，做好残疾人的康复、就业、奔小康工作。四是加强医疗卫生服务体系建设。坚持公共医疗卫生的公益性质，强化政府责任和投入，提高疾病预防控制和突发公共卫生事件应急处置能力，加强农村三级卫生服务网络和城市社区卫生服务体系建设。提高新型农村合作医疗的筹资标准和保障水平，用两年时间，把财政对参合农民的补助标准由每人每年44元提高到80元。市区和三市社区卫生服务人口覆盖率分别达到100%和70%。深化公立医院改革，强化医德医风建设，提高医疗服务质量，确保药品安全。完善计划生育利益导向机制，稳定低生育水平，提高出生人口素质。五是妥善解决中低收入群体住房问题。加快建设经济适用房、廉租房和中低价位、小户型的普通商品住宅，新开发普通商品房按不低于10%的比例配建廉租房、经济适用房，完善经济适用房销售和退出机制，扩大住房公积金覆盖面和受益面，让真正困难的群众住有所居。六是推进“平安威海”建设。加强民主法制建设，强化社会治安综合治理，完善信访工作机制，加强法律服务、援助和调解工作，发挥仲裁作用，及时防范和化解各种矛盾纠纷。强化安全生产管理和监督，有效遏制重特大安全事故。加快应急管理体系建设，完善应急指挥信息平台、预警信息发布系统，强化防灾减灾工作。加强产品质量和食品安全专项整治，整顿和规范市场经济秩序。加强社会主义精神文明建设，推进广播电视和新闻出版工作，搞好新一轮市志编修，加强档案资源的整理开发。继续做好“双拥共建”和优抚安置工作，加强国防动员、民兵预备役和人民防空建设。进一步做好妇女儿童、民族、宗教、外事、侨务、对台、气象、防震等各项工作。

各位代表！群众利益无小事。人民群众的小事，就是政府工作的大事。人民群众关心的热点、难点，就是政府工作的重点。根据社情民意调查中了解到的群众意愿，市政府确定今年着力抓好9件实事：（1）抓好米山、后龙河、院里等34座水库除险加固和母猪河下游防洪治理工程，保证人民群众生命财产安全。（2）加强城乡“双零”家庭就业援助，提高最低生活保障水平，将年人均纯收入低于1000元的农村居民、月收入低于300元的城市居民，全部纳入保障范围。（3）改造升级10个中心卫生院、40个村级卫生室，两年内将全市乡村医生轮训一遍。（4）实施政府补贴支持家电下乡，对农民购买彩电、冰箱、手机三类产品给予补贴。（5）按照每户每月400元的补贴标准扩大廉租住房制度保障范围，为低收入家庭提供5200套经济适用房。（6）对市区文化路哈工大段等10个路段的排水管网进行改造，解决路面积水问题；改造49个城中村的供排水管网，解决供水不畅、污水冒溢问题。（7）建设农家书屋180个；为全市的68个镇文化站配备新农村数字电影流动放映机，保证每村每月放映一场电影。（8）搞好农村中小学“食暖行”建设，完成40所学校食堂改造、20所学校取暖设施改造，增加学生班车25辆。（9）改造200户农村贫困残疾人危房；免费为1500名白内障盲人实施复明手术；建立工伤康复中心，加强对工伤患者的康复治疗。对这些工程，我们将强化责任，狠抓落实，不搞形式主义，努力把实事办实、好事办好，使各项工作真正体现群众的意愿、符合群众的利益、满足群众的需求。

四、进一步加快服务型政府建设

建设服务型政府是十七大提出的明确要求，也是政府职能转变的方向和重点。我们要牢记全心全意为人民服务的宗旨，坚持为民、务实、清廉，扎实推进政府自身建设和管理创新，努力做到为人民服务、对人民负责、受人民监督、让人民满意。

（一）恪守为民之责，强化服务理念。聚精会神抓项目、一心一意谋发展，改善社会民生、维护公平正义，是政府的主要职责。我们要牢记发展与和谐这两大任务，把企业的需求作为政府的职责，把人民的满意作为工作的动力。坚持“企业创造财富、政府营造环境”的理念，按照“院内的事企业干、院外的事政府办”的要求，强化主动服务意识，登门服务，现场办公，帮助企业和基层解决实际问题。充分发挥市长公开电话、行风热线、直播威海、社情民意调查中心的作用，进一步畅通与人民群众的联系渠道。对群众反映的问题和基层需要解决的困难，尽心尽力，尽职尽责，立说立行，绝不因事小而不为，切实做到件件有着落，事事有回音，给人民群众一个满意的答复。

（二）坚持依法行政，提高服务效能。深化行政审批制度改革，减少和规范行政许可和行政事业性收费，坚持多服务、少检查，多指导、少收费，多教育、少罚款，能放宽的放宽，能下放的下放，能不收的费尽量不收，降低创业门槛，减轻企业负担，方便基层办事。加强各级行政审批服务中心建设，提高集中办事程度，推动相对集中行政处罚权工作向中心镇及资源、文化、水域管理等领域延伸。落实行政执法责任制、执法过错责任追究制度和执法行为评议考核制度，严格按照宪法和法律规定的权限和程序行使权力、履行职责，着力增强政府的执行力和公信力。加强电子政务和电子监察建设，整合完善政务信息网络，提高服务效率，规范行政行为。坚持“宁可自己麻烦百次，不让群众麻烦一次”的理念，实行限时服务、承诺服务。

（三）坚持学以致用，增强服务能力。发扬理论联系实际的良好学风，认真学习十七大精神，提高运用科学理论分析和解决实际问题的能力。深入实施《公务员法》，按照五年基本轮训一遍的要求，有计划地抓好公务员培训，有针对性地开展以公共管理为核心的普及培训和现代经济、科技、社会管理等方面的专题培训，建设一支高素质、专业化的公务员队伍。加强公务员普法教育，完善学法用法制度，不断提高依法行政的能力和水平。

（四）加强作风建设，狠抓工作落实。坚持求真务实、真抓实干，进一步完善决策、执行、考核“三个体系”，每一项工作都有部署、有检查、有反馈，确保落实到位。改进会风、文风，精简会议和文件，说实话、办实事、求实效，反对形式主义、官僚主义，反对弄虚作假，不搞劳民伤财的“形象工程”、“政绩工程”，用经得起历史检验、人民评判的政绩向党和人民交一份合格的答卷。深入开展民主评议政风行风活动，严肃查处吃拿卡要、乱收乱罚、以权谋私等损害群众利益的违纪违法行为。

（五）自觉接受市委领导和人大政协监督，不断深化政务公开。工作中要紧紧依靠市委的坚强领导，从思想上、行动上坚决执行市委的各项决议和决策部署。自觉接受人大及其常委会的法律监督和政协的民主监督，接受司法监督、社会监督和舆论监督，认真听取各民主党派、工商联和人民团体的意见。完善社会公示、重大事项听证、专家决策咨询、市民旁听政府会议和市民意见建议征集等制度，保证群众充分行使知情权、参与权和监督权，提高政府的科学行政、民主行政、依法行政水平。

（六）坚持从严治政，加强廉政建设。认真践行胡锦涛同志倡导的八个方面良好风气，自觉遵守各项廉政建设规定，大力开展廉政文化建设教育活动，健全惩治和预防腐败体系，从源头上预防腐败的发生。大力提倡勤俭节约，反对奢侈浪费。认真落实“一岗双责”和行政首长问责、重要情况通报和报告、政府投资监管、经济责任审计、述职述廉、诫勉谈话等制度，加强对财政资金运行、国有资产保值增值、重大项目建设等重点环节和部位的有效监督，用制度管权、管人、管事，防止行政权力滥用，努力做到权为民所用、情为民所系、利为民所谋。

各位代表！威海是我们共同的家园，威海的发展与每一位威海人都息息相关，威海的美好未来也要靠全市人民共同创造。让我们高举中国特色社会主义伟大旗帜，紧密团结在以胡锦涛同志为总书记的党中央周围，在市委的坚强领导下，万众一心，开拓奋进，为建设繁荣富强和谐幸福的新威海而努力奋斗！
